# Bionate^®^ nucleus disc replacement: bench testing comparing two different designs

**DOI:** 10.1186/s10195-023-00692-9

**Published:** 2023-04-11

**Authors:** Amparo Vanaclocha, Vicente Vanaclocha, Carlos M. Atienza, Pablo Clavel, Pablo Jordá-Gómez, Carlos Barrios, Leyre Vanaclocha

**Affiliations:** 1 Escuela de Doctorado, Valencia, Spain; 2 Department of Surgery, University of Valencia, Valencia, Spain; 3 Instituto de Biomecánica (IBV), Universitat Politècnica de Valencia, Valencia, Spain; 4 Grupo de Tecnología Sanitaria (GTS-IBV), Instituto de Biomecánica de Valencia-CIBER BBN, Valencia, Spain; 5 Instituto Clavel, Hospital Quironsalud Barcelona, Barcelona, Spain; 6 Hospital General Universitari de Castellón, Castellón, Spain; 7 Catholic University of Valencia, Saint Vincent Martyr, Valencia, Spain

**Keywords:** Degenerative disc disease, Finite element analysis, Motion preservation, Nucleus disc replacement, Polycarbonate urethane

## Abstract

**Background:**

Intervertebral disc nucleus degeneration initiates a degenerative cascade and can induce chronic low back pain. Nucleus replacement aims to replace the nucleus while the annulus is still intact. Over time, several designs have been introduced, but the definitive solution continues to be elusive. Therefore, we aimed to create a new nucleus replacement that replicates intact intervertebral disc biomechanics, and thus has the potential for clinical applications.

**Materials and methods:**

Two implants with an outer ring and one (D2) with an additional midline strut were compared. Static and fatigue tests were performed with an INSTRON 8874 following the American Society for Testing and Materials F2267-04, F2346-05, 2077-03, D2990-01, and WK4863. Implant stiffness was analyzed at 0–300 N, 500–2000 N, and 2000–6000 N and implant compression at 300 N, 1000 N, 2000 N, and 6000 N. Wear tests were performed following ISO 18192-1:2008 and 18192-2:2010. GNU Octave software was used to calculate movement angles and parameters. The statistical analysis package R was used with the Deducer user interface. Statistically significant differences between the two designs were analyzed with ANOVA, followed by a post hoc analysis.

**Results:**

D1 had better behavior in unconfined compression tests, while D2 showed a “jump.” D2 deformed 1 mm more than D1. Sterilized implants were more rigid and deformed less. Both designs showed similar behavior under confined compression and when adding shear. A silicone annulus minimized differences between the designs. Wear under compression fatigue was negligible for D1 but permanent for D2. D1 suffered permanent height deformation but kept its width. D2 suffered less height loss than D1 but underwent a permanent width deformation. Both designs showed excellent responses to compression fatigue with no breaks, cracks, or delamination. At 10 million cycles, D2 showed 3-times higher wear than D1. D1 had better and more homogeneous behavior, and its wear was relatively low. It showed good mechanical endurance under dynamic loading conditions, with excellent response to axial compression fatigue loading without functional failure after long-term testing.

**Conclusion:**

D1 performed better than D2. Further studies in cadaveric specimens, and eventually in a clinical setting, are recommended.

*Level of evidence 2c.*

**Supplementary Information:**

The online version contains supplementary material available at 10.1186/s10195-023-00692-9.

## Introduction

Low back pain is a worldwide epidemic [1]. One of its causes is disc herniation with radicular impingement. The classical treatment, the microdiscectomy, involves removing the nucleus pulposus through a partial annulotomy. The result is a reduction in disc height and foraminal space with index level zygapophyseal joint overload causing, over time, arthritic changes and chronic low back pain [2]. The alternative is spinal fusion with vertebral segment immobilization, avoiding index-level foraminal stenosis and facet joint overload. However, the adjacent disc and its zygapophyseal joints get the extra load and mobility, inducing their degeneration long term [3]. Another option is total disc replacement, a relatively aggressive procedure requiring a retroperitoneal approach with all its risks [4].

Nucleus pulposus replacement during a discectomy is an option that attempts to prevent this degenerative cascade [5], but finding an ideal material and design is still elusive [6]. Therefore, we aimed to create a nucleus replacement inserted from a posterior approach through the annulotomy required to perform a microdiscectomy. We did an FEA (finite element analysis) of different designs and materials in a previous study, finding suitable the polycarbonate urethane (PCU) Corethane 80A, commercially known as Bionate^®^ (The Polymer Technology Group DSM-PTG, Berkeley, California, USA). This material has properties closely resembling those of the intact intervertebral disc [7].

The next step was to create a design that minimized subsidence and extrusion risks. We came up with a doughnut-shaped nucleus replacement that transmitted the load mainly to the endplate ring apophysis. But we were unsure whether this implant would be strong enough or whether a central partition should be added to increase its strength.

We present bench tests results comparing the two Bionate^®^ nucleus replacement designs.

## Material and methods

We aimed to create a new nucleus replacement that replicated intact intervertebral disc biomechanics and thus had the potential for clinical applications. This is a biomechanical laboratory bench study performed on polymeric nucleus replacements.

**Fig. 1:**
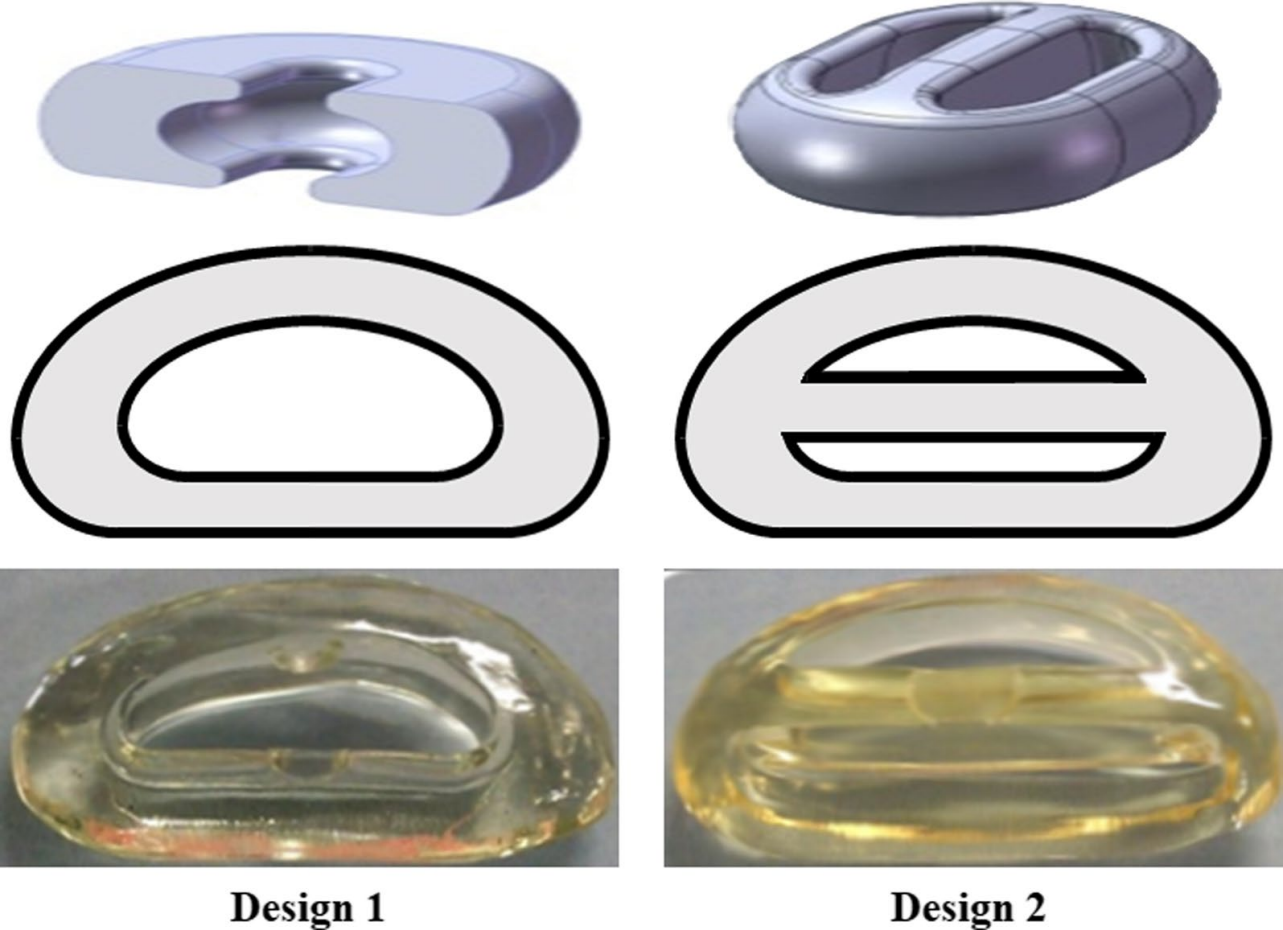
Nucleus replacement designs selected for evaluation

The material selected was Bionate^®^ 80A, with elastic modulus (E) = 22.19–23.93 MPa, and v (Poisson coefficient) = 0.4923–0.4924 [7] and elastic modulus = 22 MPa [7]. Two designs were compared (Fig. 1), both with an outer ring and one (D2) with an additional midline partition wall. Both had space in their center to allow deformation during implantation through a small annulotomy. In addition, the partition wall aimed to increase the implant’s strength.

The tests followed the ASTM (American Society for Testing and Materials) F2267-04, ASTM F2346-05, ASTM 2077-03, ASTM D2990-01, and ASTM WK4863 (Guide for Mechanical and Functional Characterization of Nucleus Devices), and were performed using the INSTRON 8874 (Canton, MA, USA), with a 25,000-N load cell. The static tests analyzed implant stiffness at 0–300 N, 500–2000 N, and 2000–6000 N and implant compression at 300 N, 1000 N, 2000 N, and 6000 N.

### Nucleus replacement unconfined compression tests

The unconfined compression tests followed the ASTM WK4863 and ASTM F2346-05 (Standard Test Methods for Static and Dynamic Characterization of Spinal Artificial Discs).

Five samples that were sterilized (25 kGy gamma radiation) and another five that were not sterilized, per implant design, were assessed, to evaluate the effects of the sterilization method on the implant’s mechanical behavior.

Each implant was placed between two metal plates aligned with the machine’s axis (Additional File 1: Fig. S1). An increasing load was applied until failure, or 6000 N was reached (the average published lumbar vertebral body compression strength [8], considered the maximum limit of the implant’s performance).

### Artificial annulus confined compression tests

An RTV 630 (GE Silicones and Adhesives, Waterford, 12188 NY, USA) silicone annulus was manufactured following the ASTM WK4863 (Standard Practice/Guide for Mechanical and Functional Characterization of Nucleus Devices), which proved successful in a previous study [9]. A 10-mm postero-lateral defect simulated the annulotomy required for the discectomy and to allow nucleus implant insertion (Additional File 2: Fig. S2).

The artificial annulus was evaluated under compression loading, considering that it should stand 500–2000 N/mm, the published estimates for a natural annulus [10].

Five artificial annulus samples were assessed between two metal compression plates aligned with the machine’s axis, and a growing compression load was applied until 6000 N was reached (Additional File 3: Fig. S3).

### Nucleus replacement confined compression tests

The nucleus replacement confined compression tests followed the ASTM WK4863 and ASTM F2346-05. Five samples per implant design previously sterilized by 25 kGy gamma radiation were evaluated. Each nucleus implant, mounted inside an artificial silicone annulus, was placed between two metal compression plates aligned with the machine’s load axis (Additional File 4: Fig. S4). A progressively higher compression load was applied until the system failed or 10,000 N was reached.

Implants were “semi-confined.” The silicone annulus was 3 mm shorter in height than the nucleus replacement so that the load was transmitted directly to the implant. In addition, the silicone ring ensured correct implant positioning between the compression plates, avoiding extrusion.

### Nucleus replacement confined compression + shear tests

The nucleus replacement confined compression and shear tests followed the ASTM WK4863 and ASTM F2346-05. Five samples per implant design previously sterilized by 25 kGy gamma radiation mounted inside the silicone artificial annulus were placed with a 17° vertical slant between the two metal plates, simulating the intact lumbar disc physiological loading conditions (Additional File 5: Fig. S5). For a 1000-N vertical load, the implant’s compression load was 956 N and the posterior-anterior shear was 292 N, a load distribution similar to that found in many everyday activities. An increasing compression load was applied until system failure, or 10,000 N was reached.

### Nucleus replacement fatigue test

The nucleus replacement fatigue tests followed the ASTM WK4863, ASTM F2346-05, and ASTM 2077-03, with a 10 million cyclic compression load of 200–1250 N at 2 Hz frequency. This load is equivalent to the physiologic compression of an intact lumbar disc during everyday activities published in in vivo studies [11–14]. Before the tests, implants were submerged in a lubricant bovine serum solution with additives to stop bacterial proliferation, and kept at 37 ± 3 °C. This lubricant is regularly used in in vitro prosthetic joint wear tests [15] (Additional File 6: Fig. S6). Five samples per implant design were evaluated under compression fatigue, and five others were used as controls with the same conditions but without load to determine the weight and dimensional changes related to the test conditions.

The sample’s weight and dimensions were measured every million cycles, and when, days after the test, they became stable. Implant wear was calculated as the weight loss between consecutive measurements while subtracting the weight increase due to the hydration measured in the control samples.

### Nucleus replacement wear performance tests

The nucleus replacement wear performance tests followed the ISO (International Organization for Standardization) 18192-1:2008 and ISO 18192-2:2010 standards and were performed using the IBV (Institute of Biomechanics of Valencia, Valencia, Spain) Spinal Disc Wear Simulator (Additional File 7: Fig. S7) under a loading mode simulating natural lumbar disc biomechanics. It allowed long-term implant endurance and wear behavior evaluation. Samples underwent combined cyclic compression and 3D motion, including flexion–extension, lateral bending, and axial torsion. Five implants were assessed under these conditions, and five other samples, used as controls, underwent the same loadings but without motion. The test lasted 10 million cycles at 2 Hz frequency. The loads applied corresponded to 50% of those used to evaluate total lumbar disc replacements (ISO 18192-1).

Implant wear was calculated every million cycles as the weight loss between consecutive measurements while subtracting the weight increase measured on the control samples. In addition, test fluid aliquots were collected for wear debris analysis and particle characterization. Deformation of the implants was also assessed, with changes in width (left-to-right), depth (front-to-back), and height (top-to-bottom) being measured every million cycles.

### Statistical analysis

We calculated descriptive statistics using Excel (Microsoft Corporation, Redmond, WA, USA) and SPSS 26 (IBM Corporation, Armonk, New York, US). We used the GNU Octave software to calculate the angles and parameters of movement (GNU General Public License, https://www.gnu.org/software/octave/index). The free software for statistical analysis R (R Development Core Team) ([16]; [17]) was used, combined with the Deducer user interface (I. Fellows, “Deducer: A Data Analysis GUI for R,” Journal of Statistical Software, vol. 49, No. 8, 2012.). The data were analyzed with an ANOVA to determine statistically significant differences among the two designs, followed by a post hoc analysis. Differences were considered to be statistically significant if *p* < 0.05.

## Results

### Results of the nucleus replacement unconfined compression tests

**Fig. 2:**
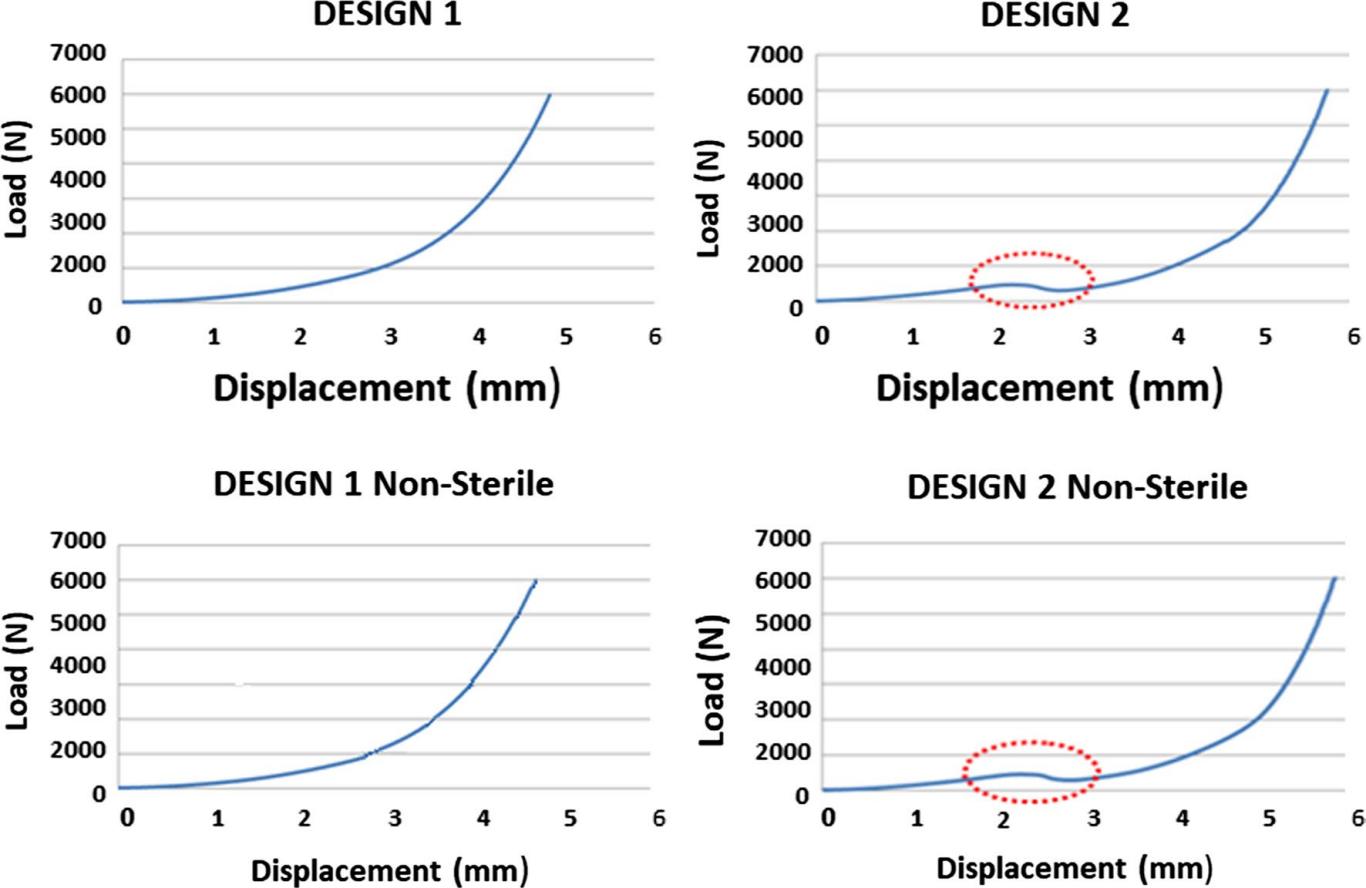
Unconfined compression load–displacement curve characteristic of each implant design

Figure 2 shows the load–displacement curve obtained during these tests. The first relevant difference between designs 1 and 2 is the inflexion point for design D2 with a 300- to 350-N compression load. This “jump” is due to the central partition wall in the implant deforming when going over 300 N.

**Fig. 3:**
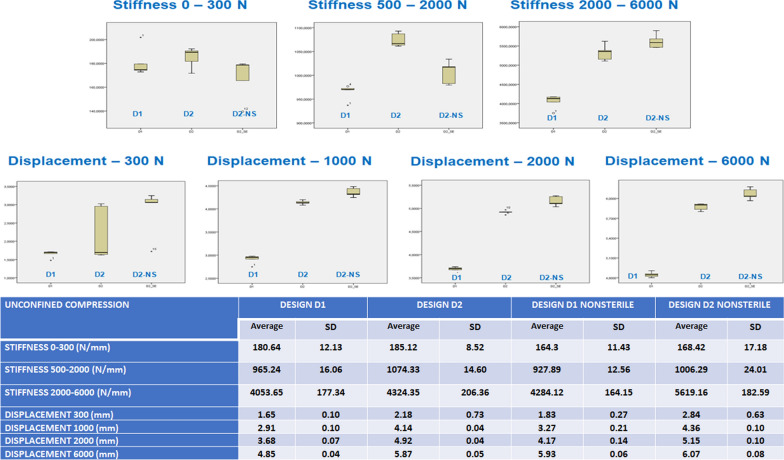
Results from the unconfined compression tests

Average and standard deviation values for both designs and non-sterilized samples regarding implant stiffness and deformation at different load ranges are presented in Fig. 3 as raw data and box and whisker plots. The data were analyzed to find any statistically significant differences between the two designs with an ANOVA followed by a post hoc analysis. The results showed that there were statistically significant differences in the mechanical behavior between both implant designs. D2 was stiffer than D1 under physiological (500–2000 N) and higher compression loads. When looking at the 25 kGy gamma sterilization influence, irradiated implants were stiffer at physiological loads (500–2000 N), while this difference was inverted for higher loads (2000–6000 N), where they showed minor deformation.

### Results for the artificial annulus confined compression tests

The annulus compression stiffness was 403.06 ± 9.94 (SD) N/mm at 0–300 N, 1116.59 ± 8.03 N/mm at 500–2000 N and 3059.89 ± 82.28 N/mm at 2000–6000 N. Thus, these data remained within the ASTM WK 4863 recommended ranges for the natural annulus under physiological loads (500–2000 N/mm) [10, 18] (Additional File 8: Fig. S8).

### Results for the nucleus replacement confined compression tests

**Fig. 4:**
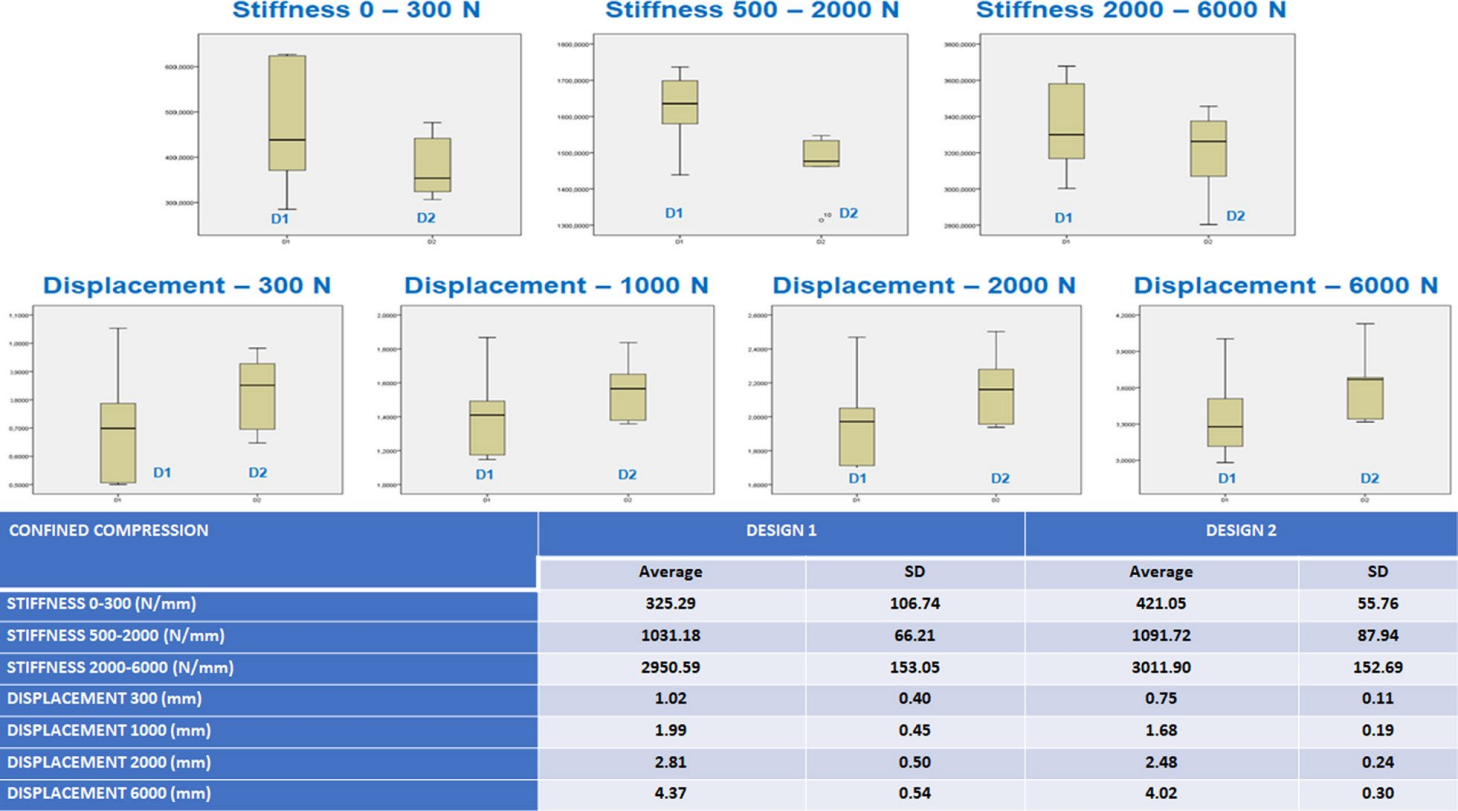
Confined compression implant stiffness and deformation under different compression loads

Both designs offered a similar behavior load–displacement curve compared with unconfined compression tests. Figure 4 presents the average and standard deviation values for the artificial disc complex (nucleus replacement + silicone annulus) stiffness and deformations at different load ranges for implant designs, represented by box and whisker plots. No statistically significant differences could be seen between the implant designs, apart from the D1 results showing higher dispersion. Furthermore, in the ANOVA analysis, no statistically significant differences were found in any parameters in the confined compression, unlike the unconfined compression.

### Results for the nucleus replacement confined compression + shear tests

**Fig. 5:**
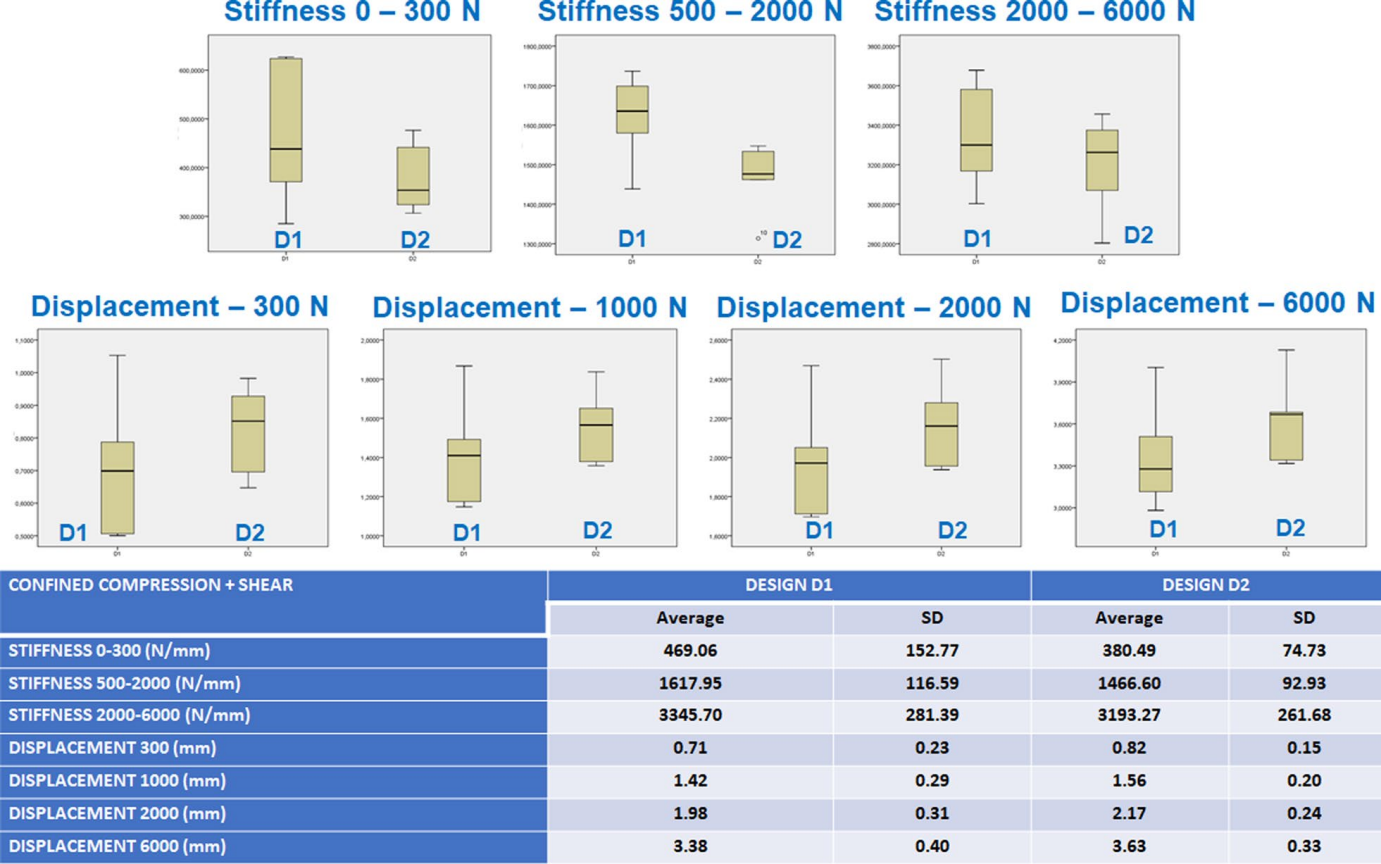
Confined compression + shear test implant stiffness and deformation under different loads

Under this test, both designs had very similar behavior. Average and standard deviation values for the artificial disc complex (nucleus implant + silicone annulus) stiffness and deformation at different load ranges and box and whisker plots are presented in Fig. 5. They show a distance between the stiffness of the two designs in the physiological load range, but no statistically significant differences were seen in the other mechanical parameters. The ANOVA analysis confirmed that both designs had different stiffness in the physiological load range (500–2000 N), but not in any of the analyzed parameters in the confined compression, probably because the silicone artificial annulus minimizes the mechanical differences between the two designs.

### Conclusions from the static strength tests

D1 had a more continuous and softer behavior under lower loads in unconfined compression tests, while D2 showed a “jump effect,” probably due to central partition wall deformation. There were statistically significant differences in stiffness and deformation between D1 and D2 under physiological and higher compression loads. D2 was stiffer than D1, and D2 deformed 1 mm more than D1. The sterilized implants were more rigid under physiological loads and deformed less.

Both designs showed equivalent behavior under confined compression, and when adding shear, D1 was stiffer in the physiological load range. In addition, the silicone annulus minimized the mechanical differences between the two designs.

### Results from fatigue tests

**Fig. 6:**
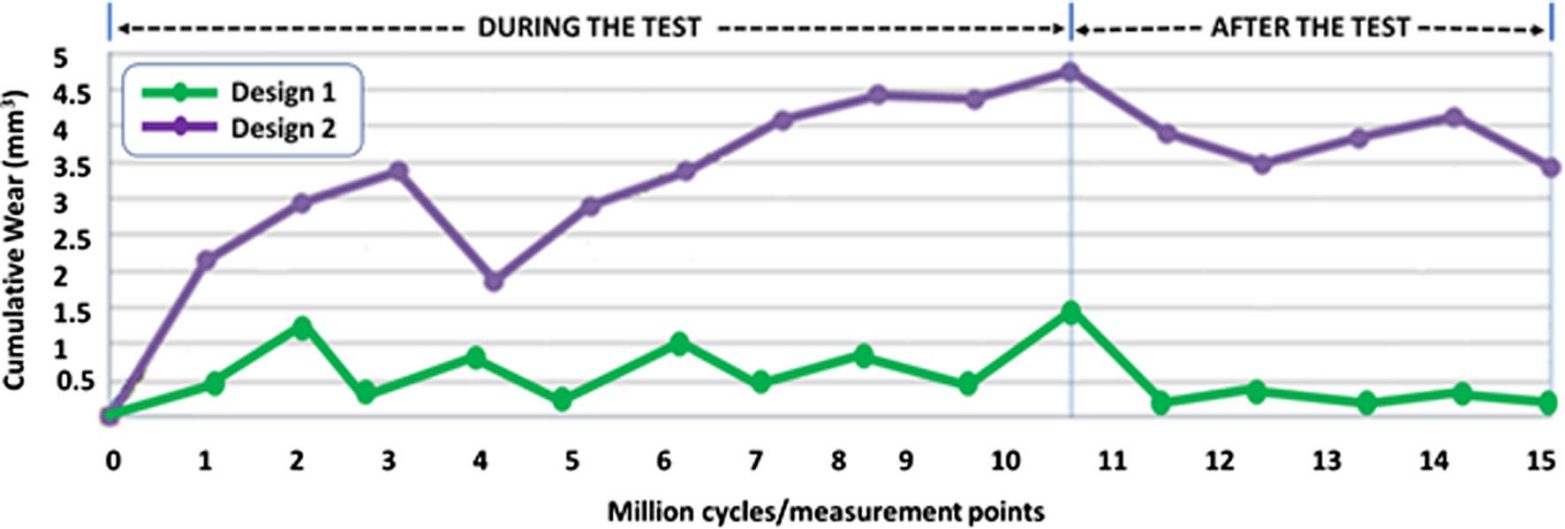
Evolution of the average cumulative volumetric wear during and after the compression fatigue test for both implant designs

The average cumulative wear measured every million cycles during the fatigue test, 4 days after it (measurement points 11–13), and 1 month later (measurement points 14–15) for both implant designs are shown in Fig. 6.

At the end of the 10 million cycles, design D1 had shrunk by an average of 1.47 mm^3^, equivalent to 0.07% of first implant volume (2144.67 mm^3^), and design D2 had shrunk by an average of 4.75 mm^3^, which is equal to 0.19% of first implant volume (2043.72 mm^3^). A few days after the test, design D1 recovered its size, and rehydrated, resulting in a complete weight rest

**Fig. 7:**
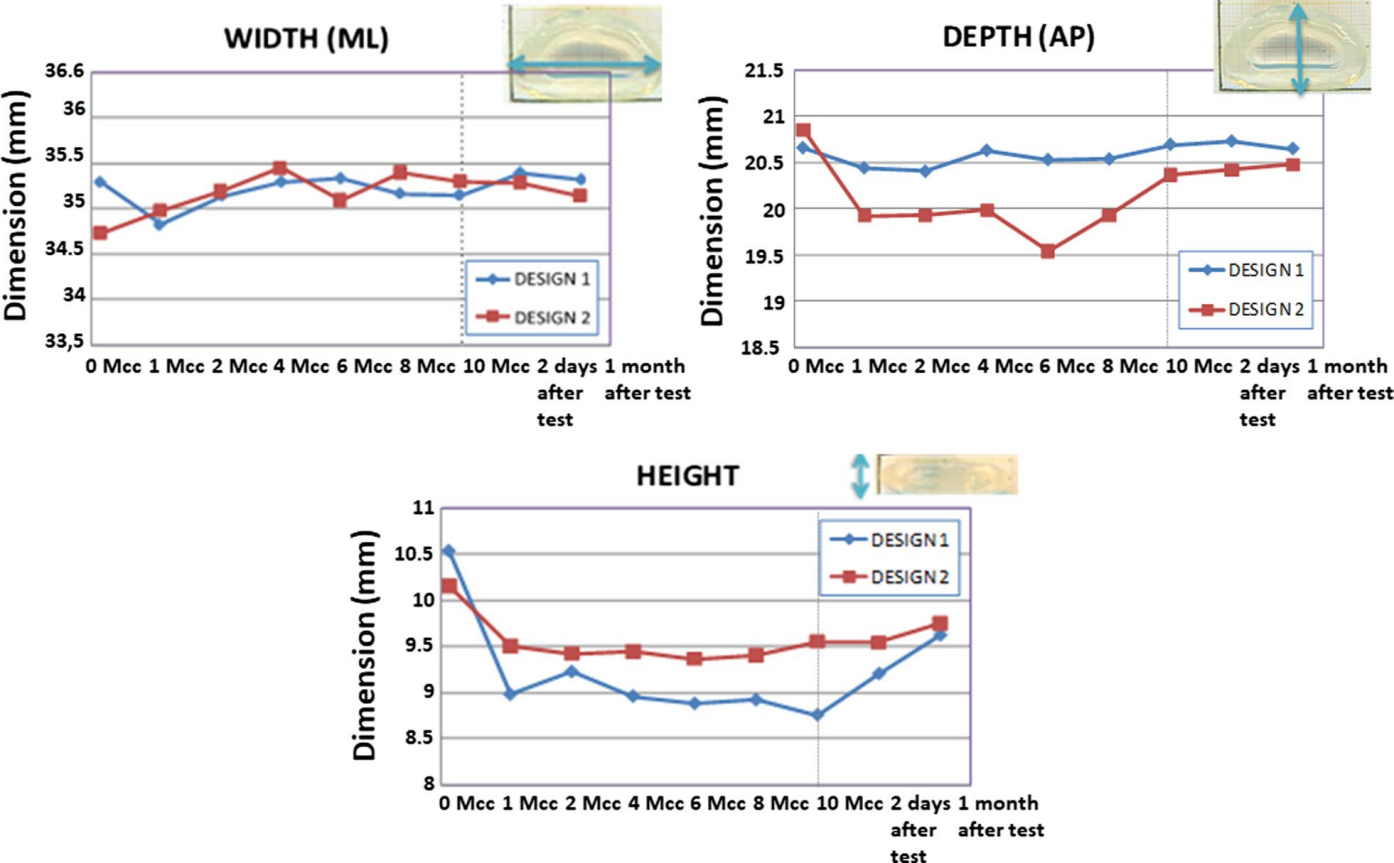
Average implant dimensions during and after fatigue tests for both designs

Besides wear, the main implant dimensions were periodically measured, looking for cyclic compression-induced permanent deformation. Every million cycles, implant samples were photographed in distinct positions using a magnifying glass and dimensions calculated with image analysis software. Figure 7 shows the evolution of the implant dimensions during and after the fatigue tests for both designs.

Under compression loading, the most affected dimension was the implant’s height, particularly for D1 (1.5 mm reduction), but D1 recovered better after it. The long-term permanent height deformation was 1 mm for D1 and 0.5 mm for D2. The side-to-side width did not significantly change for D1, while it increased 0.5 mm for D2. The anterior-posterior depth was not affected in D1, while D2 spread in that direction during the test and contracted after it. To summarize, D1 suffered a permanent deformation in height but kept its shape in the transverse plane. On the other hand, D2 suffered a minor height loss compared with D1, but it underwent a permanent deformation in the transverse plane.

### Conclusions from the fatigue test

Both designs showed excellent mechanical responses to compression fatigue with no breaks, cracks, or delamination. However, at 10 million cycles, D2 showed threefold more extensive wear than D1. After the test, hydration induced weight recovery: complete for D1 but partial for D2 due to permanent wear. Both designs lost height after fatigue compression loading and recovered it partially after the test, once unloaded. The loss was more significant for D1 than for D2, but D1 regained it better after the test. D1 did not show significant changes in depth or width at the end of the trial, while D2 was deformed in the transverse plane (0.5 mm of anterior-posterior reduction and medial-lateral augmentation). After a detailed analysis of the static and fatigue tests results, D1 was chosen for further biomechanical testing because it offered a better and more homogeneous behavior.

### D1 design wear performance test results

D1 had a tendency for an almost linear wear increase with the number of test cycles. The nucleus implant’s average volumetric wear rate was 3.22 ± 0.91 mm^3^ per million cycles (Mcc). After 7 million cycles, the average cumulative wear was 23.58 ± 7.93 mm^3^; this was 1.09% of the first implant volume, which can be considered an acceptable result (Additional File 9: Fig. S9). These results are within the range of values published for other commercial disc nuclei replacements [19–21].

**Fig. 8:**
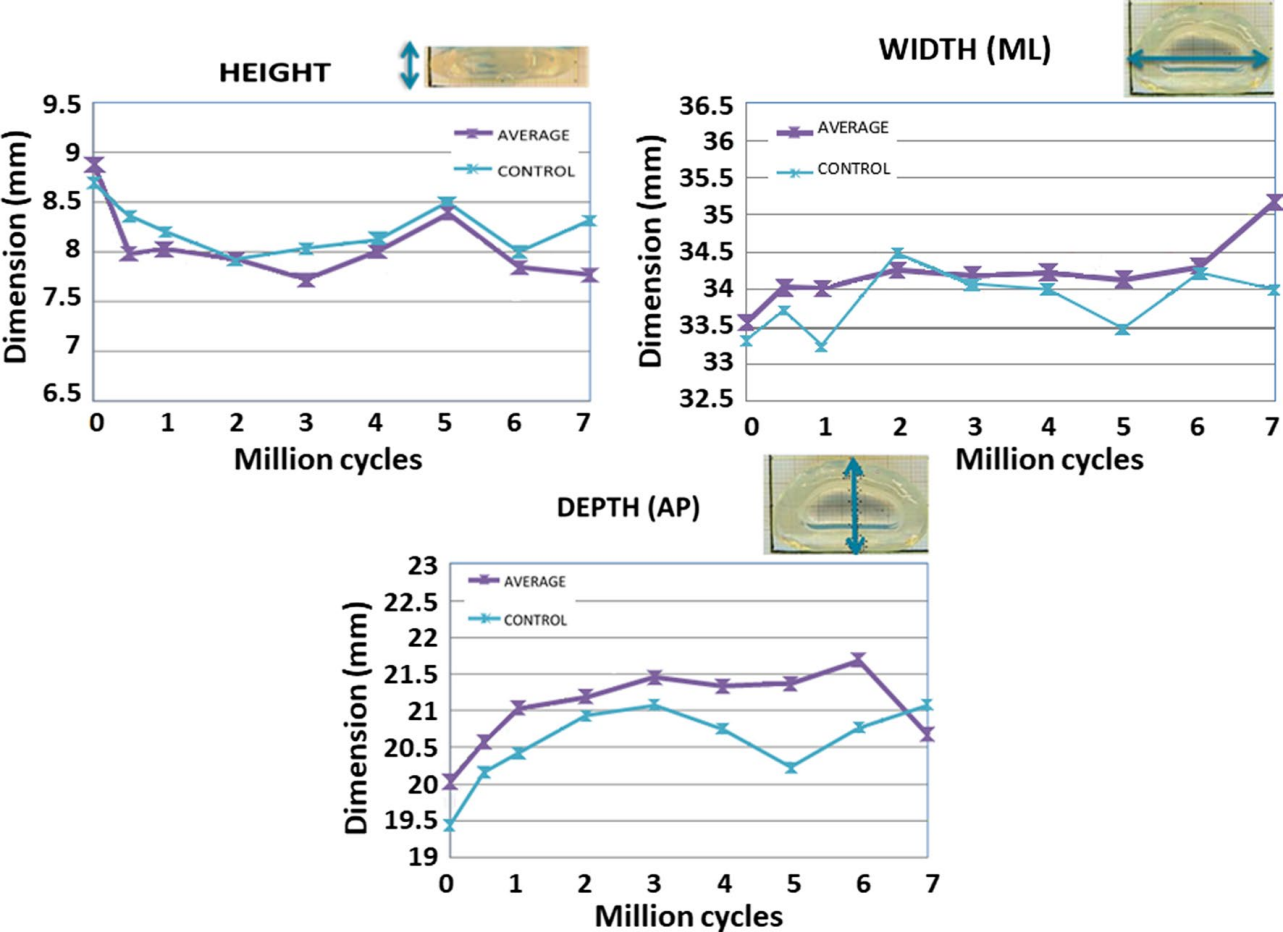
Evolution of the implant’s height, width, and depth in the wear test (up to 7 million cycles), compared with the controls (underwent the same loadings but without motion)

When looking at the effects of the wear test on the main implant dimensions, a specific permanent deformation was seen in design D1. As in the case of compression fatigue, there was a height loss (Fig. 8). The principal height reduction was produced at the beginning of the test (1 mm at 0.5 million cycles) and this was stable until 3 million cycles when there was a partial height recovery of about 0.6 mm until 5 million cycles. Then, the height decreased from 5 to 6 million cycles, with an average value of 0.6 mm. Finally, in the last million cycles, the implant height kept stable. After 7 million wear test cycles, the average implant’s height was 1 mm lower than at the start.

Dimensions in the transverse plane were also affected by wear test mechanical conditions (Fig. 8). From 0 to 6 million cycles, medial-lateral width increased about 0.75 mm. Width increase was mainly produced at the beginning of the test, after the first 0.5 Mcc, and then it kept relatively stable until 6 million cycles. Then it increased again.

Anterior-posterior (AP) depth was more affected than the side-to-side dimension. From 0 to 3 million cycles, the tendency was to increase the AP depth, with an average of 1.5 mm (Fig. 8). From 3 to 5 million cycles, the AP depth tended to keep stable. From 5 million cycles onwards, the AP dimension suffered fewer oscillations. Under physiological loading conditions, the trend was for the nucleus implant to increase the AP depth 1–2 mm and the medial-lateral width 0.5–1.5 mm.

### Conclusions from the D1 design wear test

All the tested nucleus implants showed a good mechanical response to multi-axial loading wear testing as neither breaks, cracks, nor delamination was seen in any sample. After 7 million cycles, wear suffered by the nucleus implant was relatively low, comparable to other commercial disc nuclei replacements [19–21]. However, under the wear test loading conditions, the nucleus implant suffered a permanent deformation. After 6 million cycles, the implant’s height decreased by 1 mm and the width and depth increased by 0.75 mm and 1.5 mm, respectively. Under normal physiological loading conditions, the trend is that implant dimensions change mainly initially and then keep relatively stable.

## Discussion

Nucleus replacement needs a competent annulus fibrosus with an annulotomy as small as possible [22]. Such replacement has been shown to slow the degenerative cascade aroused by the discectomy [23], but the main problems are still subsidence and extrusion [24]. The first is directly related to the implant’s rigidity and its load distribution [25]. The second problem is mainly associated with the annulotomy size, the implant design, and material properties [26].

Minimizing the subsidence demands an implant with biomechanical characteristics resembling an intact intervertebral disc [5]. Therefore, the material to be used must have properties as close as possible to the natural disc [5]. The material must have a capacity to swell with hydration and behave so that the load is not concentrated at a given point but rather distributed in a 360° fashion [27]. Furthermore, the annulus needs to be kept under appropriate tension, and the endplates must not support any of its surfaces at a higher load than it can tolerate [28]. In this respect, polycarbonate urethane is a polymer group with high resistance to pressure and good tensile strength [29], besides being biocompatible [30]. Bionate^®^ II 80A (DSM Corporate, Heerlen, Netherlands) stiffness at 1 mm displacement is 448.48 N/mm, and plastic deformation is 1.1 ± 0.2% [29]. Other materials have been tested, like cellulose [31], silicones [32], hydrogels [33], polymers [34], and alginates [35]. Although some have reached the market [23, 24], none has stood the test of time.

The second issue is how to distribute the load, and then it comes to the need for an appropriate design [36]. It is well known that the strongest point of the vertebral endplate is the ring apophysis [37]. Therefore, creating an implant that distributes the load mostly in this peripheral ring of cortical bone makes sense. That is how we came to our doughnut-shaped nucleus replacement. An additional advantage of this is that the central empty cavity and the Bionate flexibility [38] allow sideways implant compression, enabling insertion into the intervertebral space through a minimal annulotomy.

Attempting to increase the resistance of our nucleus replacement, we created a new design with a central partition wall, hoping that this would improve its performance. Unfortunately, as we have seen in our study, this was not the case, as this change was associated with more significant wear and worse biomechanical behavior. This result surprised us.

The final consideration is reduction of the extrusion risk. Indeed, a smaller annulotomy can help, but many have insisted on repairing the annulus [22]. Unfortunately, this drawback is far from being solved, as the materials used to plug the annulotomy defect do not always remain in place under the high pressures that a lumbar disc annulus must stand [39]. Nevertheless, making a soft, pliable nucleus implant certainly helps insert it through a smaller annulotomy and reduces the extrusion risk. That is why the Bionate^®^ 80A is such a good material, particularly when combined with a ring-like design.

The quest is far from over. We need to continue improving our designs and hoping that the industry will create new materials that are more compliant with nucleus disc replacement requirements.

### Limitations

The study was performed using a limited number of samples. It was a bench study with no in vivo data. Studies on human cadaveric spine specimens are needed to find out the subsidence and extrusion risks. Only two different designs with the same material were compared.

### Strengths

The implants were thoroughly tested, and the data carefully analyzed. Both weight changes and dimension changes were considered. The ANOVA and post hoc statistical analysis have allowed comparison of the two different implant designs.

## Conclusions

The Bionate^®^ confers high flexibility and compliance to the nucleus implant, minimizing breakage and cracking risk under high compression and shear loading conditions.

D1 had a better performance with minor wear but slightly higher permanent deformation than D2. Sterilization made the implants more rigid and less deformable. The artificial silicone annulus minimized the mechanical differences between the designs. Wear under compression fatigue was negligible for design D1 but permanent for design D2. Both implant designs showed an excellent response to compression fatigue with no breaks, cracks, or delamination. At 10 million cycles, D2 showed threefold more wear than D1. D1 offered a better and more homogeneous behavior, and suffered relatively low wear, comparable to other commercial disc nuclei replacements.

## Supplementary Information

**Additional file 1: Figure S1.** Unconfined compression test set-up.

**Additional file 2: Figure S2.** Silicone artificial annulus used for the confined mechanical tests with a postero-lateral 6mm defect to simulate the annulotomy.

**Additional file 3: Figure S3.** Compression test to characterize the annulus stiffness.

**Additional file 4: Figure S4.** (a) Artificial silicone annulus used for the confined mechanical tests. (b) Designs D1 and D2 were placed inside theartificial annulus. (c) The confined compression test set-up.

**Additional file 5: Figure S5.** Set-up for confined compression + shear tests.

**Additional file 6: Figure S6.** Fatigue test set-up.

**Additional file 7: Figure S7.** IBV Spinal Disc Wear Simulator applied for wear behavior assessment of the nucleus replacement devices.

**Additional file 8: Figure S8.** Silicone artificial annulus load-displacement behavior under compression load.

**Additional file 9: Figure S9.** Results from design D1 wear test.

## Data Availability

Restrictions apply to the availability of the data that support the findings of this study, because they were used under license from CUSTOM-IMD Consortium, and so are not publicly available. However, data are available from the authors upon reasonable request and with permission of the CUSTOM-IMD Consortium.

## Abbreviations

ANOVA: Analysis of variance
ASTM: American Society for Testing and Materials
FEA: Finite element analysis
IBV: Institute of Biomechanics of Valencia, Valencia, Spain
ISO: International Organization for Standardization
PCU: Polycarbonate urethane
