# Supplementary figures and images for: Bionate® nucleus disc replacement: bench testing comparing two different designs
Source: J Orthop Traumatol. 2023 Apr 11;24:13. doi: 10.1186/s10195-023-00692-9 (PMC10090247; doi:10.1186/s10195-023-00692-9)

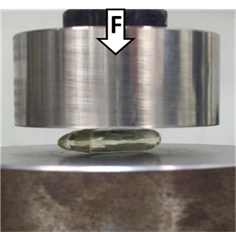

Supplement: Supplementary file 1 — Additional file 1: Figure S1. Unconfined compression test set-up. [file 10195_2023_692_MOESM1_ESM.tif]

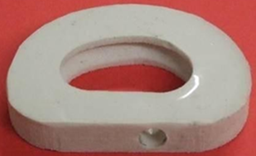

Supplement: Supplementary file 2 — Additional file 2: Figure S2. Silicone artificial annulus used for the confined mechanical tests with a postero-lateral 6mm defect to simulate the annulotomy. [file 10195_2023_692_MOESM2_ESM.tif]

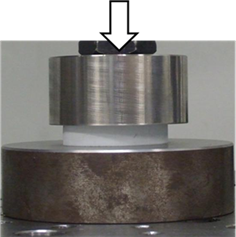

Supplement: Supplementary file 3 — Additional file 3: Figure S3. Compression test to characterize the annulus stiffness. [file 10195_2023_692_MOESM3_ESM.tif]

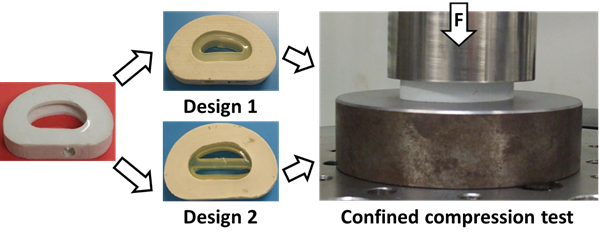

Supplement: Supplementary file 4 — Additional file 4: Figure S4. (a) Artificial silicone annulus used for the confined mechanical tests. (b) Designs D1 and D2 were placed inside theartificial annulus. (c) The confined compression test set-up. [file 10195_2023_692_MOESM4_ESM.tif]

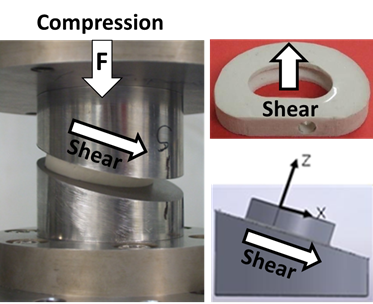

Supplement: Supplementary file 5 — Additional file 5: Figure S5. Set-up for confined compression + shear tests. [file 10195_2023_692_MOESM5_ESM.tif]

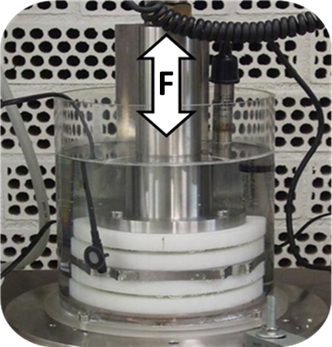

Supplement: Supplementary file 6 — Additional file 6: Figure S6. Fatigue test set-up. [file 10195_2023_692_MOESM6_ESM.tif]

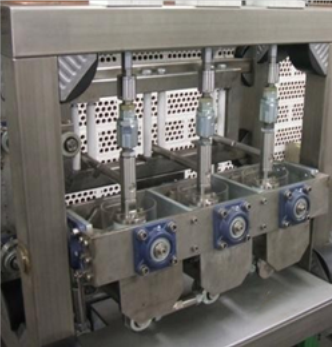

Supplement: Supplementary file 7 — Additional file 7: Figure S7. IBV Spinal Disc Wear Simulator applied for wear behavior assessment of the nucleus replacement devices. [file 10195_2023_692_MOESM7_ESM.tif]

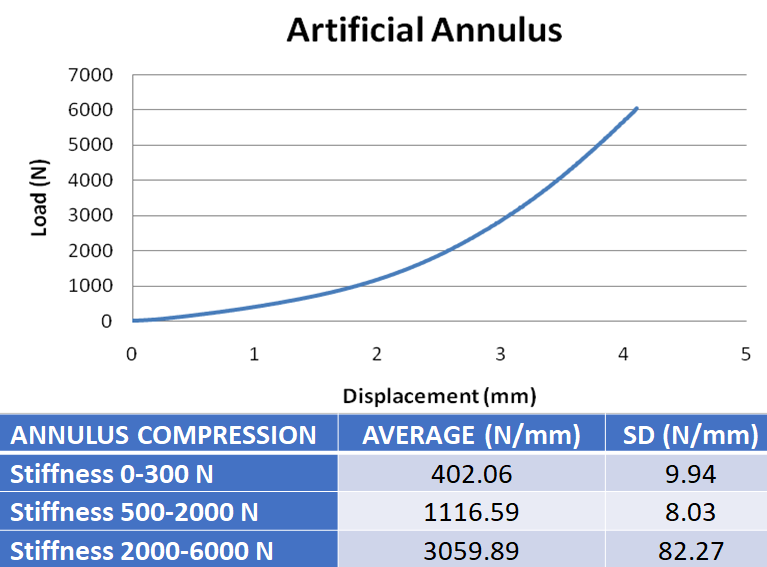

Supplement: Supplementary file 8 — Additional file 8: Figure S8. Silicone artificial annulus load-displacement behavior under compression load. [file 10195_2023_692_MOESM8_ESM.tif]

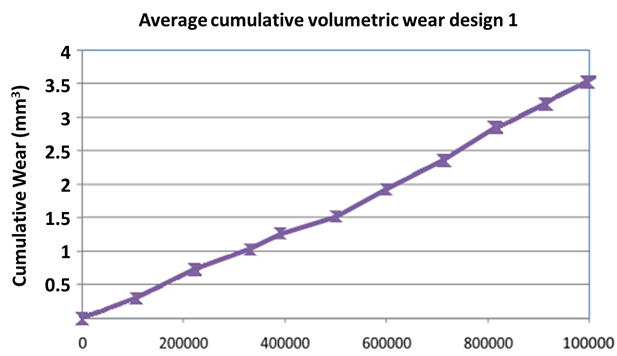

Supplement: Supplementary file 9 — Additional file 9: Figure S9. Results from design D1 wear test. [file 10195_2023_692_MOESM9_ESM.tif]
